# Developing an Improved Strategy for the Analysis of Polychlorinated Dibenzo-p-Dioxins/Furans and Dioxin-like Polychlorinated Biphenyls in Contaminated Soils Using a Combination of a One-Step Cleanup Method and Gas Chromatography with Triple Quadrupole Mass Spectrometry

**DOI:** 10.3390/toxics11090738

**Published:** 2023-08-28

**Authors:** Haena Chu, Jungmin Jo, Younggyu Son, Ji Yi Lee, Yun Gyong Ahn

**Affiliations:** 1 Western Seoul Center, Korea Basic Science Institute, Seoul 03759, Republic of Korea; haenachu9@kbsi.re.kr; 2 Department of Environmental Science and Engineering, Ewha Womans University, Seoul 03760, Republic of Korea; jjm@ewhain.net (J.J.); yijiyi@ewha.ac.kr (J.Y.L.); 3 Department of Environmental Engineering, Kumoh National Institute of Technology, Gumi 39177, Republic of Korea; yson@kumoh.ac.kr; 4 Department of Energy Engineering Convergence, Kumoh National Institute of Technology, Gumi 39177, Republic of Korea

**Keywords:** polychlorodibenzo-p-dioxins (PCDDs), polychlorodibenzofurans (PCDFs), dioxin-like polychlorinated biphenyls (dl-PCBs), persistent organic pollutants (POPs), one-step cleanup, gas chromatography with triple quadrupole mass spectrometry (GC-QqQ-MS/MS), soils

## Abstract

Soils contaminated with polychlorodibenzo-p-dioxins (PCDDs), polychlorodibenzofurans (PCDFs), and dioxin-like (dl) polychlorinated biphenyls (PCBs), known as persistent organic pollutants (POPs), have garnered global attention because of their toxicity and persistence in the environment. The standard method for target analytes has been used; however, it is an obstacle in large-scale sample analysis due to the comprehensive sample preparation and high-cost instrumental analysis. Thus, analytical development of inexpensive methods with lower barriers to determine PCDDs/Fs and dl-PCBs in soil is needed. In this study, a one-step cleanup method was developed and validated by combining a multilayer silica gel column and Florisil micro-column followed by gas chromatography with triple quadrupole mass spectrometry (GC-QqQ-MS/MS). To optimize the separation and quantification of 17 PCDDs/Fs and 12 dl-PCBs in soils, the sample cleanup and instrumental conditions were investigated. For quantification method validation, spiking experiments were conducted to determine the linearity of the calibration, recovery, and method detection limit of PCDDs/Fs and dl-PCBs using isotopic dilution GC-QqQ-MS/MS. The applicability of the simultaneous determination of PCDDs/Fs and dl-PCBs was confirmed by the recovery of native target congeners and labeled surrogate congeners spiked into the quality-control and actual soil samples. The results were in good agreement with the requirements imposed by standard methods. The findings in this work demonstrated the high accessibility of the sample cleanup and analysis methods for the efficient determination of PCDDs/Fs and dl-PCBs in contaminated soils.

## 1. Introduction

Polychlorodibenzo-p-dioxins (PCDDs) and polychlorinated dibenzofurans (PCDFs) are a group of chemical compounds that are persistent organic pollutants (POPs). Moreover, a group of polychlorinated biphenyls (PCBs), referred to as dioxin-like (dl) PCBs or coplanar PCBs, are treated as less potent versions of PCDDs/Fs due to their similar molecular structures and properties. Coplanar PCBs, including four non-*ortho*-substituted (PCB 77, 81, 126, and 169) and eight mono-*ortho*-substituted (PCB 105, 114, 118, 123, 156, 157, 167, and 189) congeners, are particularly toxic [1]. From the perspective of human exposure, 17 PCDDs/Fs and 12 dl-PCBs are considered as toxicologically concerning materials by the World Health Organization (WHO) (Table S1).

PCDDs/Fs and dl-PCBs are mainly produced through various sources, such as industrial processes, incomplete combustion of fuels, and waste incineration [2,3,4]. These compounds are considerably stable during environmental and biological degradation, resulting in their persistence in the environment and bioaccumulation in the food chain. Moreover, they cause various biological toxicities in humans, plants, and soil [5,6,7]. The most toxic congener, 2,3,7,8-tetrachlorodibenzo-para-dioxin (TCDD), exhibits severe toxicological effects, such as damage to the immune system, teratogenesis, and tumor promotion [8,9,10].

In 2004, the Stockholm Convention on POPs emphasized the control and reduction of environmental exposure to POPs, of which PCDDs/Fs and dl-PCBs are considered as representative substances. Furthermore, numerous countries have started regulating them at both the screening and action levels, as well as prohibiting their industrial discharge under Clean Water Act Effluent Guidelines [11,12]. Therefore, new analytical methods are being developed to monitor the toxicity of PCDDs/Fs and dl-PCBs in different matrices, such as water, food, soil, and air, for large-scale sample analysis.

Most studies have focused on the direct exposure of the human body to POPs through food, animal feeds, and air; however, few studies have considered the contamination and toxicity of POPs in soil. The POP concentration in soil varies depending on the type of soil; the PCDD/F and dl-PCB concentrations in soil around industrial parks and incinerators are higher than in other environmental matrices [13,14,15,16].

In Korea, the maximum residue level (MRL) for PCDDs/Fs in soil has recently been established and emission standards for dl-PCBs are being prepared [17]. The sample preparation and analytical methods for the contaminant groups, PCDDs/Fs and dl-PCBs, have already been published by authorized organizations, such as the United States Environmental Protection Agency (USEPA) [18,19,20,21]. However, these methods have limitations with respect to monitoring numerous samples from the perspective of sample cleanup and instrumental analysis. From the perspective of sample cleanup, the open-column cleanup procedure is labor-intensive and time-consuming. Moreover, this procedure requires a large space for sample preparation as well as large amounts of hazardous solvents. An automated PCDDs/Fs cleanup system overcomes the drawbacks of the manual method in terms of time and labor. However, the costs of installation (approximately $100,000) and maintenance are high. The aforementioned factors have spurred the development of more simplified, small-scale, and time-saving methods with reasonable cost, such as contaminated soil remediation technology [22,23,24,25]. For instance, the micro-column sample cleanup procedure takes a shorter time, and simplifies and reduces laboratory work when compared with the traditional manual methods, such as the USEPA method 1613 [26,27,28]. The methods proposed in this study are semiautomatic methods that can overcome the disadvantages of both traditional and automatic preparation methods. Moreover, the developed methods require a minimum workspace and reasonable cost. A gas chromatograph–high-resolution mass spectrometer (GC-HR/MS), which is mainly used for quantifying PCDDs/Fs and dl-PCBs, has excellent advantages and high sensitivity; however, methods based on GC-HR/MS incur high initial-investment and maintenance costs and require skilled operators. Furthermore, GC-HR/MS exhibits easy contamination after a high-concentration analysis, thereby requiring extreme care during the analytical procedure. Therefore, sample inspections are expensive, i.e., approximately $2000 per sample in South Korea and $500–$2500 per sample in other countries, depending on the urgency [29]. Thus, efforts are being made toward the development of alternative analytical methods. 

In a previous study, gas chromatography with triple quadrupole mass spectrometry (GC-QqQ-MS/MS) has been successfully used as an alternative and cost-effective analytical method for determining 17 PCDDs/Fs in contaminated soil [30]. This study aims to develop an analytical procedure combining a fast, miniaturized sample cleanup method and cost-effective GC-QqQ-MS/MS analysis for quantification of 17 PCDDs/Fs and 12 dl-PCBs in contaminated soil. To our knowledge, no attempt has been made to combine a fast, miniaturized sample cleanup with a GC-QqQ-MS/MS analysis technique. A simultaneous, simple, and eco-friendly cleanup method was employed using one-step cleanup columns for effectively separating POP congeners. For the GC-QqQ-MS/MS analysis, the analytical conditions were optimized for separating and quantifying PCDDs/Fs and dl-PCBs. For validating the quantitative method, spiking experiments were conducted to determine the linearity, recovery, and method detection limit (MDL) of PCDDs/Fs and dl-PCBs. The developed method could be applied for the inspection of 17 PCDDs/Fs and 12 dl-PCBs in contaminated soil, and is also expected to be used in various detection methods, such as MRL monitoring.

## 2. Materials and Methods

### 2.1. Chemical and Reagents

Standard solutions of PCDDs/Fs and PCBs congeners, namely, EPA-1613 STOCK (Native Stock Solution), EPA-1613 LCS (^13^C_12_-labeled compound stock solution), EPA-1613 ISS (internal standard solution), and EPA-1613 CVS (calibration and verification solutions, CS1–CS4) for PCDDs/Fs, and WP-STOCK, WP-LCS, WP-ISS, and WP-CVS (CS1–CS6) for dl-PCBs, were purchased from Wellington Laboratories Inc. (Guelph, ON, Canada). The organic solvents *n*-hexane and DCM and ethyl ether for pesticide residue analysis (all of analytical grade) were purchased from J.T. Baker (Phillipsburg, NJ, USA). Anhydrous sodium sulfate (Na_2_SO_4_) from FUJI-FILM Wako Chemicals USA Corporation (Richmond, VA, USA) and 95% sulfuric acid from Merck (Darmstadt, Germany) were used. The multilayer silica gel column packed in a glass tube (O.D. 6.35 mm × length 35 cm) was purchased from Supelco (Bellefonte, PA, USA). This column was composed of 3 g 10% Ag-NO_3_/silica gel, 0.9 g silica gel, 22% H_2_SO_4_/silica gel, 4.5 g 44% H_2_SO_4_/silica gel, 0.9 g silica gel, 3 g 2% KOH/silica gel, and 0.9 g silica gel. Florisil with a mesh size of 60–100 mesh purchased from Merck was used.

### 2.2. Extraction of PCDDs/Fs and dl-PCBs from Soil Samples

To perform the quality assurance and quality control (QA/QC), a pooling sample of diatomaceous earth purchased from Duksan Science (Seoul, Republic of Korea) was used as the blank and control samples without target analytes. For the actual sample analysis, the samples excavated from areas near industrial complexes with suspected soil contamination were used. The details of the soil sampling have been described in a previous study [30]. 

For method validation, 0.025–250 ng of native standards of PCDDs/Fs and dl-PCBs were spiked into accurately weighed soil (20 g). Subsequently, 1 ng of EPA-1613 LCS and 0.5 ng of WP-LCS were added to the soil samples, and 20 g of anhydrous Na_2_SO_4_ was homogenously mixed to avoid moisture. The soil samples were then sonicated for 30 min with 100 mL of acetone:*n*-hexane (1:1, *v/v*) in an ultrasonic bath (frequency = 531 kHz). The organic solvent layer was filtered through 5 g of anhydrous Na_2_SO_4_ using qualitative filter paper (Advantec^®^, Tokyo, Japan). The extract (300 mL) was collected by repeating the sonication and filtration processes thrice. The spiked extract was evaporated (Eyela, Tokyo, Japan) to remove acetone, dissolved in 100 mL *n*-hexane, and transferred into a separate funnel. The extracts were cleaned via sulfuric acid treatment before multilayer column chromatography to avoid clogging the column with sample residues because soil matrices usually have high contents of natural organic compounds, such as humic substances, lipids, pigments, and fulvic acids [31]. The extracted *n*-hexane layer was repeatedly treated with concentrated sulfuric acid until a colorless sample was obtained. The sample was then washed with 100 mL of deionized distilled water. The solvent of the extract was evaporated and concentrated under N_2_ flow and dissolved with 50 μL of nonane. Ultrasonic extraction, filtration, and concentration were similarly applied to the extraction of the actual sample performed during the validation test.

### 2.3. Sample Cleanup

To separate PCDDs/Fs and dl-PCBs from the samples, a Florisil column was used. Supelco Dioxin Prep System–Florisil Version is a manual system that consists of a multilayer silica column coupled in series with a Florisil micro-column. Florisil was preactivated overnight at 130 °C. The micro-column was filled with 0.2 g of Na_2_SO_4_ (upper layer) and 1 g of preactivated Florisil (lower layer); considerable skill is required for conducting this step. Before cleanup, the multilayer silica column was conditioned with 150 mL of *n*-hexane and the Florisil micro-column was conditioned by adding 10 mL of DCM, followed by 100 mL of *n*-hexane.

The multilayer silica gel column was then coupled over the Florisil micro-column. The concentrated extract was loaded to the top surface of the silica gel column. The first 25 mL of eluate (F1) was discarded. Consequently, 175 mL of *n*-hexane (F2–F8) and 25 mL of 2% DCM/*n*-hexane (2:98, *v/v*, F9) were collected from the connected columns for the dl-PCB fraction. For the PCDD/F fraction, 75 mL of DCM (F10–F12) was sequentially collected. Each fraction was concentrated via N_2_ gas purging and spiked with 1 ng of EPA-1613 ISS and 0.5 ng of WP-ISS. The final volume of the sample was adjusted to 50 μL using nonane and used for analysis.

### 2.4. Instrumental Analysis

GC–QqQ-MS/MS was performed using a system with a 7890B gas chromatograph coupled with a 7010 triple quadrupole mass spectrometer (Agilent Technologies, USA). The analytes were separated using a DB-5MS UI capillary column (5% diphenyl, 95% dimethyl siloxane phase, and 60 m × 0.25 mm × 0.25 μm) from J&W Scientific (Folsom, CA, USA). A 2 μL sample was injected in the splitless mode at 280 °C and 310 °C for dl-PCBs and PCDDs/Fs, respectively. A liner with a capacity of 990 μL, an inner diameter of 4 mm, and a length of 78 mm was used. For the dl-PCB analysis, the GC oven was programmed to an initial temperature of 150 °C and held at this temperature for 1 min. Thereafter, the oven was sequentially heated up to 200 °C, 260 °C, and 300 °C at 20, 2, and 10 °C/min, respectively, and held at these temperatures for 1, 4, and 10 min, respectively. For the PCDD/F analysis, the GC oven was programmed to an initial temperature of 160 °C and held at this temperature for 1 min. Subsequently, the oven was sequentially heated up to 200 °C, 220 °C, 235 °C, and 310 °C at 5, 5, 5, and 5 °C/min, respectively, and held at these temperatures for 2, 15, 5, and 20 min, respectively. High-purity helium (99.999%) was used as the carrier gas at a flow rate of 1 mL/min. The aforementioned measurements were conducted in the electron impact ionization (EI) mode. The electron emission energy was set to 70 eV, and the temperature of the ion source was 230 °C. The analytical conditions and each specification of the instruments are summarized in Table S2. 

Each analyte was detected by selecting two specific ions (M and M+2 or M+2 and M+4) and was qualitatively analyzed by comparing the ion abundance ratio and retention time of the selected ions [32]. The quantification of the analytes was conducted using an isotopic dilution method under the dynamic MRM (dMRM) mode; this method is a good alternative for the simultaneous analysis of multicomponent samples and exhibits higher sensitivity and specificity than those of MRM [33,34]. The MRM transitions for the quantifier and qualifier utilized the two most predominant fragments for each analyte. Four individual collision energies (5, 15, 30, and 50 eV) were tested for each MRM transition. The optimized conditions for the dMRM for dl-PCBs and PCDDs/Fs are listed in Table 1.

### 2.5. Quality Assurance and Quality Control (QA/QC)

QA/QC procedures were performed to evaluate whether the developed analytical method meets the requirements of EPA 1613B and EPA 1668C methods in this study [35]. Linearity, MDL, limit of quantitation (LOQ) and recovery experiments of the calibration curve were performed to evaluate the QA/QC of the assay method. Peaks detected at a signal-to-noise ratio greater than 3 were used for the analysis. The average relative response factor (RRF) for the individual isomers was calculated at each concentration and averaged. The RSD of the average RRF value was <15%, which satisfies the EPA criterion. The linearity of the calibration curve was evaluated based on the determination coefficient (R^2^).

To assess the instrument performance and calibration during the continuous analysis, a recovery (%) test was performed by analyzing the native and ^13^C-labeled congeners with the mid-standard solution of the calibration curve for every 10 sample batches. The results showed that the recovery of the native and ^13^C-labeled PCDDs/Fs and dl-PCBs congeners satisfies the criteria of the EPA 1613B [18] and EPA 1668C [19] methods (Figure S1). Thus, GC-QqQ-MS/MS remained stable during the aforementioned analysis.

## 3. Results and Discussion

### 3.1. Optimization of the Cleanup Procedure

In the USEPA method, two columns (multilayer silica gel column and alumina column) are separately prepared in parallel, and the eluate obtained from the first column (silica gel) is concentrated. Subsequently, the eluate is newly loaded in the second column (alumina) for separation and elution. The adopted cleanup method is a one-step method that connects two columns (multilayer silica gel and Florisil column, Figure S2). Moreover, it can separate and elute PCDDs/Fs and dl-PCBs simultaneously with one loading. In this study, operational conditions such as the amount and speed of elution and the ratio of the solvent were optimized for this purpose.

Soil contamination by PCDDs/Fs and dl-PCBs are attributed to accidental spillage during the manufacture, transport, storage, and use of various chlorinated compounds in the industry as well as their disposal in unregulated landfills [36]. The concentrations of other interferences are considerably higher than those of the target analytes in contaminated soil; therefore, sample cleanup is required to remove the interferences prior to analysis and quantification. Among the sample cleanup methods, open-column absorption chromatography is commonly used for sorbents, such as silica, alumina, and Florisil [13,37,38]. In this study, a combination of multilayer silica gel and Florisil micro-columns was employed after sulfuric acid treatment. The multilayer silica gel column is used to remove interferences, including acidic and basic compounds as well as hydrolyzed fats. Furthermore, the Florisil column, which is effective in terms of recovery and resolution of dl-PCBs [39], is used for separating PCDDs/Fs and dl-PCBs with different retentions [40]. The conditions of the combined cleanup columns, namely, the specific elution solvent and flow rate, should be adjusted to the enable separation of two contaminant groups (PCDDs/Fs and dl-PCBs) in one step. 

The elution properties of dl-PCBs and PCDDs/Fs with respect to the multilayer silica gel column were investigated to determine the amounts of *n*-hexane required to elute all congeners from the silica gel column and transfer them to the coupled Florisil micro-column (Figure S3). Because higher-chlorinated biphenyl (CB) has lower polarity, hepta-CB was eluted first with *n*-hexane, a non-polar solvent, and tetra-CBs were eluted last from the silica gel column. For the same reason, OCDD and OCDF were eluted first and TCDD and TCDF were eluted last with *n*-hexane [41,42]. It was found that 25 mL of *n*-hexane was to be discarded and 175 mL of *n*-hexane was required to elute all of the congeners of dl-PCBs and PCDDs/Fs from the silica gel column.

**Figure 1:**
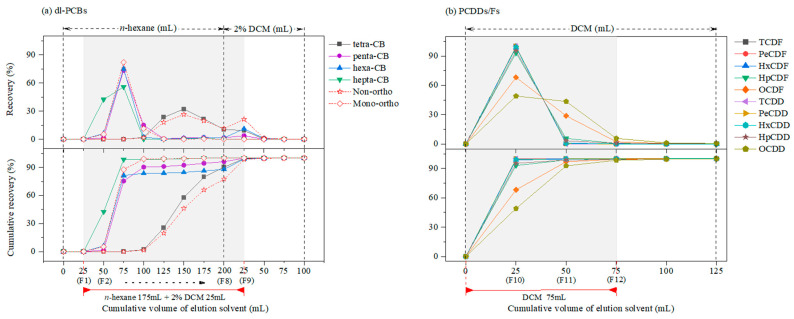
Elution profiles of (**a**) dl-PCBs and (**b**) PCDDs/Fs using the combined columns (multilayer silica gel and Florisil micro-column). The shaded part of the graph represents the elution volumes for dl-PCB fraction eluted by 175 mL of *n*-hexane and 25 mL of 2% dichloromethane (DCM) in *n*-hexane and PCDD/F fraction eluted by 75 mL of DCM.

When congeners are fractionated using the combined columns, the elution profile (Figure 1a) shows that the non-*ortho*-substituted PCBs (PCB 77, 81, 126, and 169) tend to have structurally higher retention in Florisil than the mono-*ortho*-substituted PCBs (PCB 105, 114, 118, 123, 156, 157, 167, and 189) because of their planar conformation [43]. Penta-, hexa-, and hepta-CBs were eluted from F2 to F4 with *n*-hexane, and tetra-CBs are eluted from F5 to the next elution with 25 mL of 2% dichloromethane (DCM) in *n*-hexane (F9). The elution of most hexa- and penta-CBs was not observed in more than 100 mL of *n*-hexane (Figure S4). To elute the remaining congeners, DCM/*n*-hexane (2:98 and 5:95, *v/v*) and ethyl ether/*n*-hexane (6:94, *v/v*) were considered, based on previous studies [44,45,46], as shown in Table S3. There is no considerable difference in the recovery of dl-PCBs according to the solvent composition in the Florisil micro-column; however, the highest recoveries of 92.1%–102.9% for all congeners were noted when DCM/*n*-hexane (2:98, *v/v*) was used. Figure 1b shows the elution profile of PCDDs/Fs for the Florisil micro-column with DCM after eluting dl-PCBs. As shown in Figure 1b, 75 mL of DCM (F10–F12) was required to elute all the congeners of PCDDs/Fs.

The elution rate of DCM/*n*-hexane (2:98, *v/v*) in the Florisil micro-column is a key factor for separately collecting dl-PCB and PCDDs/Fs fractions. Table 2 shows the recoveries of dl-PCBs and PCDDs/Fs when DCM/*n*-hexane (2:98, *v/v*) was eluted at a rate of 1, 5, and 10 mL/min in the Florisil micro-column. The recoveries of the dl-PCBs congeners are similar at the three different elution rates, except for PCB 169 (75.1% at a rate of 10 mL/min). Non-*ortho*-substituted PCBs (PCB 77, 81, 126, and 169) were coeluted with the PCDDs/Fs congeners at elution rates of 5 and 10 mL/min. The recoveries of the PCDDs/Fs congeners noticeably decreased with increasing elution rate from 1 and 5 mL/min to 10 mL/min. Therefore, 1 mL/min DCM/*n*-hexane (2:98, *v/v*) and DCM in the Florisil micro-column were considered the most appropriate conditions for the complete separation of the fractions of dl-PCBs and PCDDs/Fs. The specific details of each step are presented in Figure S2.

### 3.2. Method Validation

The entire analytical procedure from the sample preparation to the final analysis was validated in accordance with the linearity of calibration, MDL, LOQ, and recovery. Calibration curves of the 17 PCDDs/Fs and 12 dl-PCBs were plotted using the isotope dilution method with ^13^C-labeled standards. The determination coefficient (R^2^) exhibits good linearity of more than 0.9999 over the 0.05–200 pg/µL range for 12 dl-PCBs. For the PCDDs/Fs, the R^2^ values were in the range of 0.9992–1 for different congener concentrations, as shown in Table 3. The MDL was measured by analyzing seven replicate samples spiked with 12.5 pg of dl-PCBs and 10–50 pg of PCDDs/Fs per 20 g control sample, which is considered free of the target analytes. MDL is defined as the lowest concentration of the dl-PCBs and PCDDs/Fs congeners, resulting in a confidence of >99% when the entire analytical procedure was conducted [47]. LOQ is defined as 10 times the standard deviation (SD) obtained using MDL. Although not the same conditions, MDL obtained through comparable previous studies were summarized and compared (Table S4). Since different approaches were used to obtain MDLs by these previous studies, comparisons with this study along the same lines are limited. Among them, MDLs for PCDDs/Fs in fly ash measured by Fan et al. (2017) using the same approach were similar to those measured in this study [48]. Even though the sample matrix and the methods of extraction and cleanup are different, it is noteworthy that the analytical instrument used by Fan et al. (2017) was an expensive and highly specialized HRGC-HRMS, rather than GC-QqQ-MS/MS used in this study. Therefore, it was found that the developed analytical method is an effective alternative method in terms of cost effectiveness for the analysis of dl-PCBs and PCDDs/Fs.

The accuracy and precision values were assessed by recovery experiments using triplicate QC samples spiked with low, medium, and high concentrations of native 17 PCDDs/Fs and 12 dl-PCBs in this study. The analytical results of the samples used for recovery that were spiked with designated concentrations are shown in Table 3. The overall recoveries ranged from 87.1%~109.0% for 17 PCDDs/Fs, and 83.5%~106.5% for 12 dl-PCBs. The precisions of dl-PCBs and PCDDs/Fs were determined as the relative standard deviation (%RSD), which was calculated from the average and the standard deviation of the replicate analysis. Both the accuracy and RSD imposed by the QA/QC guidelines were satisfied. 

**Figure 2:**
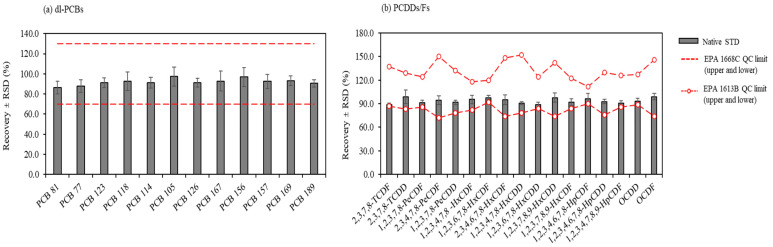
Precision and accuracy of native (**a**) dl-PCBs and (**b**) PCDDs/Fs under the optimum conditions of sample preparation obtained by the recovery assays. The bars represent the mean recoveries of the native congeners spiked into the control samples (*n* = 9). The error bars indicate the relative standard deviation (RSD). The red dots represent the upper and lower QC limits imposed by EPA 1668C and EPA 1613B methods.

As shown in Figure 2, the precision and accuracy of the targeted individual PCDDs/Fs and dl-PCBs in this study are in good agreement with the QC acceptance limits imposed by the USEPA method [49].

### 3.3. Application to Actual Soil Samples

**Figure 3:**
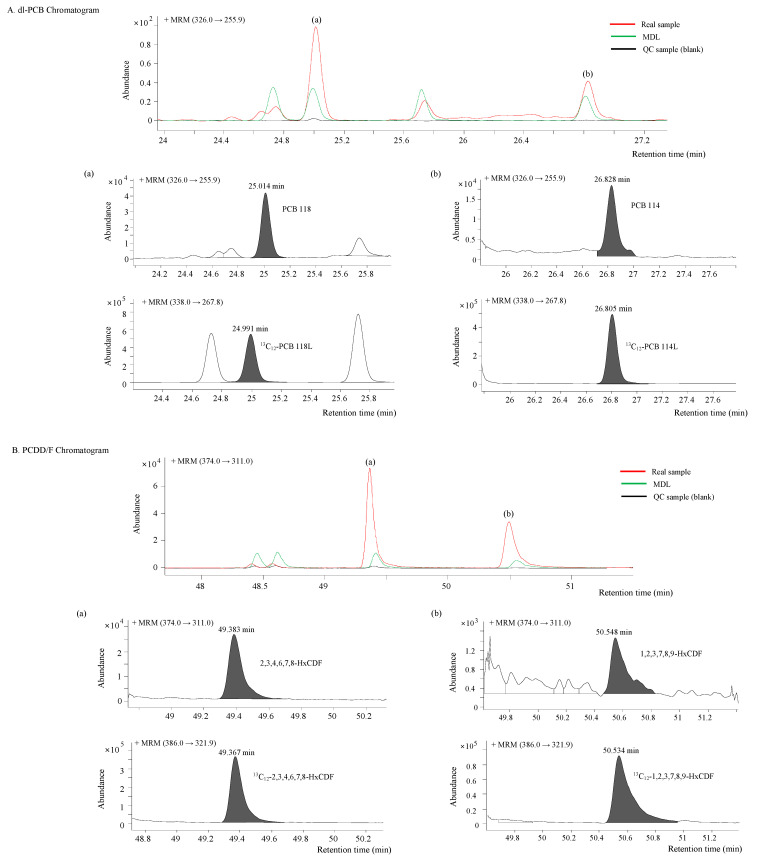
Overlay signals of the MRM chromatograms obtained by a blank, experimental MDL, and positive soil sample; (**A**) dl-PCB Chromatogram (**a**) 1.59 pg/g 2,3′,4,4′,5-pentachlorobiphenyl (PCB 118) and its ^13^C labeled standard, and (**b**) 0.50 pg/g 2,3,4,4′,5-pentachlorobiphenyl (PCB 114) and its ^13^C labeled standard, (**B**) PCDD/F Chromatogram (**a**) 2.88 pg/g 2,3,4,6,7,8-hexachlorodibenzofuran (HxCDF) and its ^13^C labeled standard, and (**b**) 0.65 pg/g 1,2,3,7,8,9-HxCDF and its ^13^C labeled standard.

The developed analytical method combined with the one-step cleanup for the separation and quantification of 17 PCDDs/Fs and 12 dl-PCBs based on GC-QqQ-MS/MS was applied to the actual soil samples. Figure 3 shows a typical overlaid MRM chromatogram to compare the signals of dl-PCBs and PCDDs/Fs detected in the soil samples excavated near industrial complexes (red color) with the blank (black color) and the signal at the lowest quantifiable concentration (MDL, green color). Although dl-PCBs (1.59 pg/g for PCB 118 and 0.50 pg/g for PCB 114) are detected at extremely low concentrations, both native and label congeners are not affected by the baseline separation and interfering substances. Moreover, for PCDDs/Fs, 2.88 pg/g 2,3,4,6,7,8-HxCDF and 0.65 pg/g 1,2,3,7,8,9-HxCDF are successfully separated from any interferences at the baseline level of the actual sample. 

**Figure 4:**
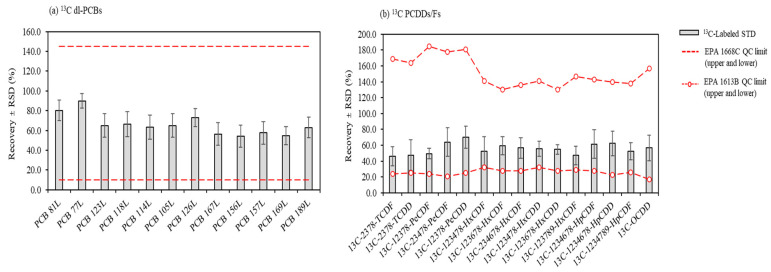
Average recoveries of ^13^C labeled (**a**) dl-PCBs and (**b**) PCDDs/Fs congeners.

Figure 4 shows the average recoveries of the spiked ^13^C-labeled surrogates of dl-PCBs and PCDDs/Fs before the sample preparation for sample analysis. The average recoveries of the labeled standards are in the range of 54.2%–90.0% with an RSD of 15% for 12 ^13^C-dl-PCBs and 46.1%–69.9% with an RSD of 20% for 15 ^13^C-PCDDs/Fs. These results satisfy the QC criteria of the EN 16190:2018 method (50%–130% for tetrachlorinated to hexachlorinated congeners and 40%–130% for heptachlorinated and octachlorinated congeners) [50,51,52]. The sample cleanup and instrumental analysis were confirmed to be performed under the appropriate cleanup efficiency and quantitative conditions. 

To determine the residue levels of PCDDs/Fs in the environmental media for regulation purposes, the total toxic equivalent (TEQ) value based on the WHO toxicity equivalence factor (TEF) rather than the concentration of a single congener is commonly used. The total TEQ concentration value is the sum of the values obtained by multiplying the concentration of each congener by its assigned WHO-TEF. The analytical results of the representative sample with the most diverse dl-PCBs and PCDDs/Fs congeners are determined among the samples, as presented in Table S5.

More than 90% of human exposure to PCDDs/Fs and dl-PCBs originates from food, which has the lowest governmental regulatory concentration restriction among different media [53,54]. In the case of soil, the regulation level differs depending on the country or land use. In Korea, an acceptable standard of 100 pg TEQ/g has been set for PCDDs/Fs. Considering various references, the analytical method developed in this study is sufficiently applicable because the exposure limits of PCDDs/Fs and dl-PCBs in soil are in the ranges of 2.48–39,300 [55,56,57,58] and 0.05–8.8 pg TEQ/g [36,59,60], respectively.

**Figure 5:**
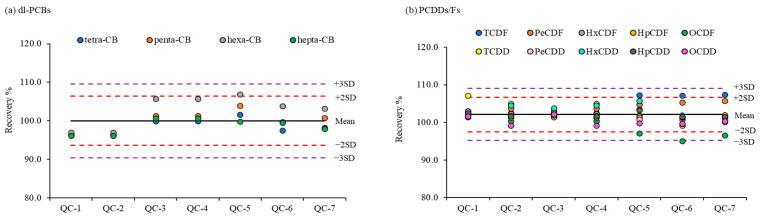
QC results obtained during sample analysis for each homologue group (tetra-, penta-, hexa-, hepta-, and octa-) of (**a**) dl-PCBs and (**b**) PCDDs/Fs. The total recoveries of each homologue group for dl-PCBs and PCDDs/Fs were calculated using the sum of individual congeners of the same degree of chlorination. The dotted lines represent 95% and 99% confidence intervals (±2SD and ±3SD, respectively).

QC samples prepared by spiking dl-PCBs and PCDDs/Fs in the middle calibration range were analyzed during sample analysis. The QC charts used to validate the analytical results obtained for every batch during sample analysis are shown in Figure 5.

Based on their degree of chlorination, 12 dl-PCBs and 17 PCDDs/Fs were categorized into homologue groups. The recoveries for each homologue group were calculated from the total concentrations of their corresponding congeners. The QC charts were constructed from the measured mean recoveries and SD of each homologue group of dl-PCBs and PCDDs/Fs for seven QC samples during sample analysis. The confidence intervals of 95% and 99% were established based on the SD values of mean ± 2SD and ±3SD, respectively [61]. Although several homologues of the PCDDs/Fs were outside the 99% confidence interval, all results were within the 95% confidence interval.

## 4. Conclusions

A simple and cost-effective GC-QqQ-MS/MS combined with a one-step cleanup method was optimized and evaluated for the simultaneous determination of 17 PCDDs/Fs and 12 dl-PCBs in contaminated soils. For the cleanup and separation of PCDDs/Fs and dl-PCBs, the elution conditions, such as solvent composition and elution flow rate, of the multilayer silica gel column connected with a Florisil micro-column were optimized. For optimizing the quantitative analysis with GC-QqQ-MS/MS, the dMRM mode was used to obtain improved separation and sensitivity than MRM. To evaluate the entire analytical procedure, the linearity of the calibration curve, MDL, and recovery for the target dl-PCBs and PCDDs/Fs were validated. Results proved the validity of the proposed method, which satisfies the requirements of standard official methods. The proposed method is expected to increase the accessibility of PCDDs/Fs and dl-PCBs analyses at the laboratory scale, thereby promoting the active monitoring for their regulation and broadening the scope of related research.

## Figures and Tables

**Table 1:** Dynamic multiple-reaction monitoring (dMRM) conditions of (a) dioxin-like (dl) polychlorinated biphenyls (PCBs) and (b) polychlorodibenzo-p-dioxin (PCDD) and polychlorinated dibenzofuran (PCDF) congeners using gas chromatography with triple quadrupole mass spectrometry (GC-QqQ-MS/MS).

Congener	Qualifier		Quantifier		Congener (Labeled)	Qualifier		Quantifier	
	Transitions (*m/z*)	Collision Energy (eV)	Transitions (*m/z*)	Collision Energy (eV)		Transitions (*m/z*)	Collision Energy (eV)	Transitions (*m/z*)	Collision Energy (eV)
(a) dl-PCBs									
PCB 81	290.0 → 220.1	30	292.0 → 222.0	30	PCB 81L	302.0 → 232.1	30	304.0 → 234.0	30
PCB 77	290.0 → 219.9	30	292.0 → 222.0	30	PCB 77L	302.0 → 232.1	30	304.0 → 234.0	30
PCB 123	328.0 → 257.9	30	326.0 → 255.9	30	PCB 123L	340.0 → 270.0	30	338.0 → 267.8	30
PCB 118	328.0 → 257.9	30	326.0 → 255.9	30	PCB 118L	340.0 → 270.0	30	338.0 → 267.8	30
PCB 114	328.0 → 257.9	30	326.0 → 255.9	15	PCB 114L	340.0 → 270.0	30	338.0 → 267.8	30
PCB 105	328.0 → 257.9	30	326.0 → 255.9	30	PCB 105L	340.0 → 270.0	30	338.0 → 267.8	30
PCB 126	328.0 → 257.9	30	326.0 → 255.7	30	PCB 126L	340.0 → 269.9	30	338.0 → 267.9	30
PCB 167	362.0 → 291.9	30	360.0 → 289.8	30	PCB 167L	374.0 → 303.9	30	372.0 → 301.9	30
PCB 156	362.0 → 291.9	30	360.0 → 290.0	30	PCB 156L	374.0 → 303.8	15	372.0 → 301.8	30
PCB 157	362.0 → 291.9	15	360.0 → 290.0	30	PCB 157L	374.0 → 303.8	30	372.0 → 301.8	30
PCB 169	362.0 → 291.9	30	360.0 → 289.7	30	PCB 169L	374.0 → 303.8	30	372.0 → 302.0	30
PCB 189	396.0 → 325.9	30	394.0 → 324.1	30	PCB 189L	408.0 → 338.0	30	406.0 → 336.0	30
					PCB 70L	302.0 → 232.0	30	304.0 → 233.9	30
					PCB 111L	340.0 → 269.8	30	338.0 → 267.8	30
					PCB 138L	374.0 → 303.9	30	372.0 → 301.9	15
					PCB 170L	408.0 → 338.0	30	406.0 → 336.0	30
(b) PCDDs/Fs									
2,3,7,8-TCDF	304.0 → 241.0	30	306.0 → 242.9	30	^13^C-2,3,7,8-TCDF	316.0 → 252.3	30	318.0 → 253.9	50
2,3,7,8-TCDD	320.0 → 256.8	50	322.0 → 193.9	50	^13^C-2,3,7,8-TCDD	332.0 → 268.1	15	334.0 → 269.8	15
1,2,3,7,8-PeCDF	342.0 → 278.8	30	340.0 → 276.9	30	^13^C-1,2,3,7,8-PeCDF	354.0 → 290.2	15	352.0 → 287.9	30
2,3,4,7,8-PeCDF	342.0 → 278.9	30	340.0 → 276.9	30	^13^C-2,3,4,7,8-PeCDF	354.0 → 289.7	30	352.0 → 287.9	30
1,2,3,7,8-PeCDD	358.0 → 294.9	30	356.0 → 229.9	50	^13^C-1,2,3,7,8-PeCDD	370.0 → 306.1	15	368.0 → 303.9	15
1,2,3,4,7,8-HxCDF	376.0 → 313.0	30	374.0 → 311.0	30	^13^C-1,2,3,4,7,8-HxCDF	388.0 → 324.1	15	386.0 → 321.9	30
1,2,3,6,7,8-HxCDF	376.0 → 313.0	30	374.0 → 311.0	30	^13^C-1,2,3,6,7,8-HxCDF	388.0 → 324.2	15	386.0 → 321.9	15
2,3,4,6,7,8-HxCDF	376.0 → 312.9	30	374.0 → 311.0	30	^13^C-2,3,4,6,7,8-HxCDF	388.0 → 324.0	30	386.0 → 321.9	30
1,2,3,4,7,8-HxCDD	392.0 → 329.0	15	390.0 → 264.0	50	^13^C-1,2,3,4,7,8-HxCDD	404.0 → 276.0	30	402.0 → 273.9	50
1,2,3,6,7,8-HxCDD	392.0 → 329.0	15	390.0 → 264.0	50	^13^C-1,2,3,6,7,8-HxCDD	404.0 → 276.1	30	402.0 → 273.9	50
1,2,3,7,8,9-HxCDD	392.0 → 329.0	15	390.0 → 264.0	50	^13^C-1,2,3,7,8,9-HxCDF	388.0 → 323.8	15	386.0 → 321.8	30
1,2,3,7,8,9-HxCDF	376.0 → 312.9	30	374.0 → 310.9	30	^13^C-1,2,3,7,8,9-HxCDF	388.0 → 323.8	15	386.0 → 321.8	30
1,2,3,4,6,7,8-HpCDF	410.0 → 347.0	30	408.0 → 344.9	30	^13^C-1,2,3,4,6,7,8-HpCDF	422.0 → 357.8	50	420.0 → 356.1	30
1,2,3,4,6,7,8-HpCDD	426.0 → 361.0	30	424.0 → 363.0	30	^13^C-1,2,3,4,6,7,8-HpCDD	438.0 → 373.9	30	436.0 → 372.0	30
1,2,3,4,7,8,9-HpCDF	410.0 → 346.9	30	408.0 → 345.0	30	^13^C-1,2,3,4,7,8,9-HpCDF	422.0 → 358.1	30	420.0 → 356.0	30
OCDD	458.0 → 395.0	30	460.0 → 397.0	15	^13^C-OCDD	470.0 → 406.1	30	472.0 → 408.2	30
OCDF	442.0 → 378.9	30	444.0 → 381.0	30	^13^C-OCDD	470.0 → 406.1	30	472.0 → 408.2	30
					^13^C-1,2,3,4-TCDD	332.0 → 267.9	15	334.0 → 269.8	15
					^13^C-1,2,3,7,8,9-HxCDD	404.0 → 276.1	30	402.0 → 273.9	50

**Table 2:** Recoveries ^a^ (%) of dl-PCBs and PCDDs/Fs congeners based on the elution rate (mL/min) in the Florisil micro-column.

Congener	1 mL/min		5 mL/min		10 mL/min	
	dl-PCB Fraction ^b^	PCDD/F Fraction ^c^	dl-PCB Fraction	PCDD/F Fraction	dl-PCB Fraction	PCDD/F Fraction
dl-PCBs						
PCB 81	100.0	ND ^d^	99.8	0.2	98.9	1.1
PCB 77	100.0	ND	99.6	0.4	95.8	4.2
PCB 123	100.0	ND	99.9	0.1	100.0	ND
PCB 118	100.0	ND	99.9	0.1	100.0	ND
PCB 114	100.0	ND	100.0	ND	100.0	ND
PCB 105	100.0	ND	99.6	0.4	99.8	0.2
PCB 126	100.0	ND	99.4	0.6	92.8	7.2
PCB 167	100.0	ND	99.9	0.1	100.0	ND
PCB 156	100.0	ND	99.9	0.1	100.0	ND
PCB 157	100.0	ND	99.9	0.1	100.0	ND
PCB 169	100.0	ND	97.9	2.1	75.1	24.9
PCB 189	100.0	ND	99.9	0.1	100.0	ND
PCDDs						
2,3,7,8-TCDD	ND	87.2	ND	77.8	ND	30.6
1,2,3,7,8-PeCDD	ND	98.2	ND	86.1	ND	28.1
1,2,3,4,7,8-HxCDD	ND	106.0	ND	110.7	ND	31.2
1,2,3,6,7,8-HxCDD	ND	97.2	ND	98.6	ND	26.8
1,2,3,7,8,9-HxCDD	ND	101.6	ND	115.0	ND	26.7
1,2,3,4,6,7,8-HpCDD	ND	107.4	ND	107.7	ND	31.4
OCDD	ND	98.1	ND	88.2	ND	26.3
PCDFs						
2,3,7,8-TCDF	ND	89.0	ND	84.1	ND	35.8
1,2,3,7,8-PeCDF	ND	101.4	ND	83.2	ND	30.6
2,3,4,7,8-PeCDF	ND	111.5	ND	100.2	ND	31.6
1,2,3,4,7,8-HxCDF	ND	90.8	ND	83.7	ND	23.5
1,2,3,6,7,8-HxCDF	ND	109.4	ND	94.8	ND	32.0
2,3,4,6,7,8-HxCDF	ND	107.4	ND	100.5	ND	27.1
1,2,3,4,6,7,8-HpCDF	ND	105.4	ND	100.4	ND	30.1
1,2,3,4,7,8,9-HpCDF	ND	116.5	ND	97.4	ND	30.7
OCDF	ND	99.3	ND	92.9	ND	27.3

^a^ Recovery of dl-PCBs means the amount recovered from each fraction (dl-PCB and PCDD/F fractions) when the total amount of all fractions was 100% recovery. Recovery of PCDDs/Fs means the percentage of a measured concentration relative to the spiked concentration. ^b^ dl-PCB fraction: F2-F9 as shown in Figure S4. ^c^ PCDD/F fraction: F10–F12 as shown in Figure S4. ^d^ ND: Not detected.

**Table 3:** Calibration, method detection limit (MDL), limit of quantification (LOQ) and recoveries of target (a) dl-PCBs and (b) PCDDs/Fs.

Congener	Calibration		Avg. RRF	MDL ^a^ (pg/g)	LOQ ^b^ (pg/g)	Low Conc. QC Sample	Medium Conc. QC Sample			High Conc. QC Sample	
	Conc. Range (pg/µL)	R^2^				Conc. (pg/g)	Recovery ± RSD (%)	Conc. (pg/g)	Recovery ± RSD (%)	Conc. (pg/g)	Recovery ± RSD (%)
(a) dl-PCBs											
PCB 81	0.05–200	0.9999	1.0706	0.258	0.823	1.25	86.4 ± 12.8	125	83.5 ± 3.2	1250	88.9 ± 3.2
PCB 77	0.05–200	0.9999	1.0809	0.161	0.513	1.25	92.0 ± 11.4	125	86.7 ± 2.6	1250	85.4 ± 3.1
PCB 123	0.05–200	0.9999	1.0281	0.222	0.708	1.25	96.5 ± 4.4	125	86.9 ± 0.8	1250	90.1 ± 3.0
PCB 118	0.05–200	0.9999	1.1090	0.233	0.741	1.25	104.3 ± 4.5	125	88.8 ± 1.6	1250	85.2 ± 3.6
PCB 114	0.05–200	0.9999	0.9170	0.211	0.671	1.25	93.1 ± 7.9	125	86.3 ± 1.3	1250	93.8 ± 3.1
PCB 105	0.05–200	0.9999	0.8921	0.375	1.195	1.25	106.5 ± 9.7	125	94.9 ± 6.7	1250	90.7 ± 3.3
PCB 126	0.05–200	0.9999	1.0234	0.229	0.729	1.25	94.1 ± 4.3	125	86.3 ± 1.1	1250	92.6 ± 2.9
PCB 167	0.05–200	0.9999	0.9669	0.218	0.694	1.25	105.2 ± 6.7	125	85.8 ± 2.8	1250	87.6 ± 3.2
PCB 156	0.05–200	0.9999	1.0720	0.331	1.054	1.25	104.2 ± 11.5	125	88.7 ± 3.6	1250	97.8 ± 2.9
PCB 157	0.05–200	0.9999	1.0565	0.289	0.920	1.25	99.1 ± 6.1	125	84.9 ± 0.4	1250	93.7 ± 3.0
PCB 169	0.05–200	0.9999	0.8749	0.313	0.996	1.25	90.9 ± 6.7	125	93.5 ± 5.6	1250	95.3 ± 3.7
(b) PCDDs/Fs											
2,3,7,8-TCDF	0.05–40	1.0000	2.1009	0.228	0.727	12.5	88.6 ± 1.8	125	90.4 ± 3.2	1250	87.6 ± 0.2
2,3,7,8-TCDD	0.1–40	0.9998	0.5316	0.322	1.026	1.25	109.0 ± 0.8	12.5	97.3 ± 1.9	125	90.1 ± 4.9
1,2,3,7,8-PeCDF	0.25–200	1.0000	0.9809	0.215	0.684	62.5	95.4 ± 1.0	125	89.9 ± 0.2	1250	88.3 ± 0.4
2,3,4,7,8-PeCDF	0.25–200	1.0000	1.1026	1.016	3.236	12.5	92.6 ± 1.7	625	89.8 ± 1.2	6250	101.1 ± 4.4
1,2,3,7,8-PeCDD	0.25–200	0.9999	1.1355	0.273	0.868	62.5	94.5 ± 1.8	625	89.7 ± 0.8	1250	91.3 ± 3.4
1,2,3,4,7,8-HxCDF	0.25–200	0.9998	1.3921	0.761	2.425	62.5	93.8 ± 1.0	625	91.0 ± 0.9	6250	101.5 ± 3.8
1,2,3,6,7,8-HxCDF	0.25–200	1.0000	1.3647	0.849	2.704	62.5	98.2 ± 2.3	625	95.6 ± 1.3	6250	99.2 ± 3.8
2,3,4,6,7,8-HxCDF	0.25–200	0.9998	1.2599	1.071	3.41	62.5	92.8 ± 2.4	625	89.8 ± 1.3	6250	102.1 ± 4.5
1,2,3,4,7,8-HxCDD	0.25–200	0.9999	1.0625	0.251	0.799	12.5	90.8 ± 2.9	125	90.0 ± 1.1	1250	91.4 ± 2.7
1,2,3,6,7,8-HxCDD	2.5–200	1.0000	0.9712	0.862	2.744	12.5	89.3 ± 2.7	125	89.4 ± 6.9	1250	87.1 ± 2.0
1,2,3,7,8,9-HxCDD	0.25–200	0.9992	1.8255	1.045	3.327	12.5	95.7 ± 7.4	125	103.3 ± 4.3	1250	94.3 ± 2.5
1,2,3,7,8,9-HxCDF	0.25–200	1.0000	0.8419	0.527	1.678	12.5	91.2 ± 4.0	125	96.3 ± 1.6	1250	88.5 ± 3.3
1,2,3,4,6,7,8-HpCDF	0.25–200	1.0000	0.9889	1.112	3.543	125	92.9 ± 1.1	1250	91.3 ± 1.1	12,500	105.3 ± 2.9
1,2,3,4,6,7,8-HpCDD	0.25–200	0.9999	0.9120	1.423	4.531	125	92.9 ± 1.7	1250	91.3 ± 0.9	12,500	94.2 ± 4.7
1,2,3,4,7,8,9-HpCDF	0.25–200	0.9997	0.8585	0.626	1.993	12.5	93.2 ± 2.6	125	90.8 ± 1.8	1250	87.8 ± 4.3
OCDD	0.5–400	0.9999	1.0493	0.571	1.819	125	97.2 ± 2.8	1250	92.2 ± 0.7	12,500	90.2 ± 2.5
OCDF	0.5–400	0.9992	1.1692	0.853	2.718	125	101.0 ± 3.0	1250	95.3 ± 1.9	12,500	100.8 ± 4.9

^a^ MDL = SD × t _(n−1, 1−α = 0.99)_, t (6, 0.99) = 3.14 (*n* = 7). ^b^ LOQ = SD × 10.

## Data Availability

The data presented in this study are available on request from the corresponding author.

## Supplementary Materials

The following supporting information can be downloaded at: https://www.mdpi.com/article/10.3390/toxics11090738/s1, Table S1. Physicochemical properties and toxic equivalent factors of target dl-PCBs and PCDDs/Fs; Table S2. Instrumental conditions for the quantification of dl-PCBs and PCDDs/Fs; Table S3. Recoveries (%) of dl-PCB congeners according to different elution solvent compositions; Table S4. Comparison of MDLs obtained by different extraction, cleanup, and detection methods in the previous studies with the ones in this study; Table S5. Total WHO-TEQ concentrations of dl-PCBs and PCDDs/Fs for the representative soil samples excavated near industrial complexes; Figure S1. Verification charts of (a) dl-PCBs and (b) PCDDs/Fs obtained during sample analysis to monitor the instrumental performance (GC-QqQ-MS/MS); Figure S2. Schematic of the one-step cleanup procedure; Figure S3. Elution profiles of (a) dl-PCBs and (b) PCDDs/Fs on the multilayer silica gel column; Figure S4. Chromatograms of congeners of dl-PCBs and PCDDs/Fs obtained during fractionation. References [62,63,64,65,66,67,68] are cited in the Supplementary Materials.
