# Supplementary material for: Developing an Improved Strategy for the Analysis of Polychlorinated Dibenzo-p-Dioxins/Furans and Dioxin-like Polychlorinated Biphenyls in Contaminated Soils Using a Combination of a One-Step Cleanup Method and Gas Chromatography with Triple Quadrupole Mass Spectrometry
Source: Toxics. 2023 Aug 28;11(9):738. doi: 10.3390/toxics11090738 (PMC10536111; doi:10.3390/toxics11090738)
Supplement: Supplementary file 1 [file toxics-11-00738-s001.zip › toxics-2540383-supplementary.docx]

Supplementary Materials

Developing an Improved Strategy for the Analysis of Polychlorinated Dibenzo-p-Dioxins/Furans and Dioxin-Like Polychlorinated Biphenyls in Contaminated Soils Using a Combination of a One-Step Cleanup Method and Gas Chromatography with Triple Quadrupole Mass Spectrometry

Haena Chu ^1,†^, Jungmin Jo ^2,†^, Younggyu Son ^3,4^, Ji Yi Lee ^2^ and Yun Gyong Ahn ^1,^*

Contents:

**Table S1**. Physicochemical properties and toxic equivalent factors of target dl-PCBs and PCDDs/Fs.

**Table S2.** Instrumental conditions for the quantification of dl-PCBs and PCDDs/Fs.

**Table S3.** Recoveries (%) of dl-PCB congeners according to different elution solvent compositions.

**Table S4.** Comparison of MDLs obtained by different extraction, cleanup, and detection methods in the previous studies with the ones in this study.

**Table S5.** Total WHO-TEQ concentrations of dl-PCBs and PCDDs/Fs for the representative soil sample excavated near industrial complexes.

**Figure S1.** Verification charts of (a) dl-PCBs and (b) PCDDs/Fs obtained during sample analysis to monitor the instrumental performance (GC-QqQ-MS/MS).

**Figure S2.** Schematic of the one-step clean-up procedure.

**Figure S3.** Elution profiles of (a) dl-PCBs and (b) PCDDs/Fs on the multilayer silica gel column.

**Figure S4.** Chromatograms of congeners of dl-PCBs and PCDDs/Fs obtained during fractionation.

**Table S1.** Physicochemical properties and toxic equivalent factors of target dl-PCBs and PCDDs/Fs.

| **Classification** | **Congener** | **CAS No.** | **Molecular**  **formula** | **Molecular**  **weight (g/moL)** | **Water solubility**  **(mg/L at 25°C)^a^** | **log K_ow_^b^** | **WHO_2005_ TEF ^c^** |
| --- | --- | --- | --- | --- | --- | --- | --- |
| Non-*ortho*-substituted PCBs | |  |  |  |  |  |  |
|  | 3,3',4,4'-Tetrachlorobiphenyl (PCB 77) | 32598-13-3 | C_12_H_6_Cl_4_ | 292.0 | 1.0×10^–3^ | 6.5 | 0.0001 |
|  | 3,4,4',5-Tetrachlorobiphenyl (PCB 81) | 70362-50-4 | C_12_H_6_Cl_4_ | 292.0 | 2.92×10^–3^ | 6.36 | 0.0003 |
|  | 3,3',4,4',5-Pentachlorobiphenyl (PCB 126) | 57465-28-8 | C_12_H_5_Cl_5_ | 326.4 | 1.03×10^–3^ | 6.89 | 0.1 |
|  | 3,3',4,4',5,5'-Hexachlorobiphenyl (PCB 169) | 32774-16-6 | C_12_H_4_Cl_6_ | 360.9 | 3.61×10^–5^ | 7.49 | 0.03 |
|  | |  |  |  |  |  |  |
| Mono-*ortho*-substituted PCBs | |  |  |  |  |  |  |
|  | 2,3,3',4,4'-Pentachlorobiphenyl (PCB 105) | 32598-14-4 | C_12_H_5_Cl_5_ | 326.4 | 1.90×10^–3^ | 6.0 | 0.00003 |
|  | 2,3,4,4',5-Pentachlorobiphenyl (PCB 114) | 74472-37-0 | C_12_H_5_Cl_5_ | 326.4 | 2.58×10^–3^ (20°C) | 6.65 | 0.00003 |
|  | 2,3',4,4',5-Pentachlorobiphenyl (PCB 118) | 31508-00-6 | C_12_H_5_Cl_5_ | 326.4 | 1.59×10^–3^ (20°C) | 7.12 | 0.00003 |
|  | 2',3,4,4',5-Pentachlorobiphenyl (PCB 123) | 65510-44-3 | C_12_H_5_Cl_5_ | 326.4 | 1.64×10^–3^ | 6.74 | 0.00003 |
|  | 2,3,3',4,4',5-Hexachlorobiphenyl (PCB 156) | 38380-08-4 | C_12_H_4_Cl_6_ | 360.9 | 4.10×10^–4^ (20°C) | 7.16 | 0.00003 |
|  | 2,3,3',4,4',5'-Hexachlorobiphenyl (PCB 157) | 69782-90-7 | C_12_H_4_Cl_6_ | 360.9 | 3.61×10^–4^ | 7.19 | 0.00003 |
|  | 2,3',4,4',5,5'-Hexachlorobiphenyl (PCB 167) | 52663-72-6 | C_12_H_4_Cl_6_ | 360.9 | 3.61×10^–5^ | 7.09 | 0.00003 |
|  | 2,3,3',4,4',5,5'-Heptachlorobiphenyl (PCB 189) | 39635-31-9 | C_12_H_3_Cl_7_ | 395.3 | 6.26×10^–5^ | 7.71 | 0.00003 |
|  | |  |  |  |  |  |  |
| Polychlorinated Dibenzo-para(p)-dioxins (PCDDs) | | |  |  |  |  |  |
|  | 2,3,7,8-Tetrachlorodibenzo-p-dioxin (2,3,7,8-TCDD) | 1746-01-6 | C_12_H_4_Cl_4_O_2_ | 322.0 | 1.93×10^–5^ | 6.80 | 1 |
|  | 1,2,3,7,8-Pentachlorodibenzo-p-dioxin (1,2,3,7,8-PeCDD) | 40321-76-4 | C_12_H_3_Cl_5_O_2_ | 356.4 | - | 6.64 | 1 |
|  | 1,2,3,4,7,8-Hexachlorodibenzo-p-dioxin (1,2,3,4,7,8-HxCDD) | 39227-28-6 | C_12_H_2_Cl_6_O_2_ | 390.9 | 4.42×10^–6^ (20°C) | 7.80 | 0.1 |
|  | 1,2,3,6,7,8-Hexachlorodibenzo-p-dioxin (1,2,3,6,7,8-HxCDD) | 57653-85-7 | C_12_H_2_Cl_6_O_2_ | 390.9 | - | - | 0.1 |
|  | 1,2,3,7,8,9-Hexachlorodibenzo-p-dioxin (1,2,3,7,8,9-HxCDD) | 19408-74-3 | C_12_H_2_Cl_6_O_2_ | 390.9 | - | - | 0.1 |
|  | 1,2,3,4,6,7,8-Heptachlorodibenzo-p-dioxin (1,2,3,4,6,7,8-HpCDD) | 35822-46-9 | C_12_HCl_7_O_2_ | 425.3 | 2.40×10^–6^ (20°C) | 8.00 | 0.01 |
|  | Octachlorodibenzo-p-dioxin (OCDD) | 3268-87-9 | C_12_Cl_8_O_2_ | 459.7 | 7.40×10^–8^ | 8.20 | 0.0003 |
|  | |  |  |  |  |  |  |
| Polychlorinated Dibenzofurans (PCDFs) | |  |  |  |  |  |  |
|  | 2,3,7,8-Tetrachlorodibenzofuran (2,3,7,8-TCDF) | 51207-31-9 | C_12_H_4_Cl_4_O | 306.0 | 4.19×10^–4^ (22.7°C) | 6.1 | 0.1 |
|  | 1,2,3,7,8-Pentachlorodibenzofuran (1,2,3,7,8-PeCDF) | 57117-41-6 | C_12_H_3_Cl_5_O | 340.4 | - | 6.79 | 0.03 |
|  | 2,3,4,7,8-Pentachlorodibenzofuran (2,3,4,7,8-PeCDF) | 57117-31-4 | C_12_H_3_Cl_5_O | 340.4 | 2.36×10^–4^ (22.7°C) | 6.5 | 0.3 |
|  | 1,2,3,4,7,8-Hexachlorodibenzofuran (1,2,3,4,7,8-HxCDF) | 70648-26-9 | C_12_H_2_Cl_6_O | 374.9 | 8.25×10^–6^ (22.7°C) | 7.0 | 0.1 |
|  | 1,2,3,6,7,8-Hexachlorodibenzofuran (1,2,3,6,7,8-HxCDF) | 57117-44-9 | C_12_H_2_Cl_6_O | 374.9 | 1.77×10^–4^ (22.7°C) | - | 0.1 |
|  | 1,2,3,7,8,9-Hexachlorodibenzofuran (1,2,3,7,8,9-HxCDF) | 72918-21-9 | C_12_H_2_Cl_6_O | 374.9 | - | - | 0.1 |
|  | 2,3,4,6,7,8-Hexachlorodibenzofuran (2,3,4,6,7,8-HxCDF) | 60851-34-5 | C_12_H_2_Cl_6_O | 374.9 | - | - | 0.1 |
|  | 1,2,3,4,6,7,8-Heptachlorodibenzofuran (1,2,3,4,6,7,8-HpCDF) | 67562-39-4 | C_12_HCl_7_O | 409.3 | 1.35×10^–6^ (22.7°C) | 7.4 | 0.01 |
|  | 1,2,3,4,7,8,9-Heptachlorodibenzofuran (1,2,3,4,7,8,9-HpCDF) | 55673-89-7 | C_12_HCl_7_O | 409.3 | - | - | 0.01 |
|  | Octachlorodibenzofuran (OCDF) | 39001-02-0 | C_12_Cl_8_O | 443.7 | 1.16×10^–6^ | 8.0 | 0.0003 |

^a^ [62] and [63]**^,^** ^b^ log K_ow_; Octanol–water partition coefficient [63], ^c^ [7]

**Table S2.** Instrumental conditions for the quantification of dl-PCBs and PCDDs/Fs.

| **Instrument** | **GC-QqQ-MS/MS**  **(Agilent 7890B Gas Chromatography 7010 Triple Quad MSD)** |
| --- | --- |
| Column | DB-5MS UI (60 m × 0.25 mm × 0.25 μm) |
| Gas (gas flow) | He (carrier gas, 1 mL/min), N_2_ (collision gas, 1.5 mL/min) |
| Injector  (mode/Temp/Vol) | Splitless mode / 280 ˚C (dl-PCBs) and 310 ˚C (PCDDs/Fs) / 2uL |
| Detector | EI mode : 70eV (electron energy), 230 ˚C (ion source temp.) |
| Acquisition mode | dMRM mode |
| Oven program | dl-PCBs;  150 °C (hold for 1 min) → 200 °C (20 °C/min, hold for 1 min) → 260 °C (2 °C/min, hold for 4 min) → 300 °C (10 °C/min, hold for 10 min)  PCDDs/Fs;  160 °C (hold for 1 min) → 200 °C (5 °C/min, hold for 2 min) → 220 °C (5 °C/min, hold for 15 min) → 235 °C (5 °C/min, hold for 5 min) → 310 °C (5 °C/min, hold for 20 min) |

**Table S3.** Recoveries (%) of dl-PCB congeners according to different elution solvent compositions.

| **Congener** | **DCM/*n*-hexane (2:98, v/v)** | **DCM/*n*-hexane( 5:95, v/v)** | **Ether/*n*-hexane (6:94, v/v)** |
| --- | --- | --- | --- |
| PCB 81 | 100.9 | 92.8 | 102.9 |
| PCB 77 | 99.1 | 109.0 | 95.0 |
| PCB 123 | 99.2 | 103.6 | 99.0 |
| PCB 118 | 100.4 | 103.1 | 94.1 |
| PCB 114 | 102.1 | 101.7 | 97.6 |
| PCB 105 | 102.9 | 102.2 | 105.0 |
| PCB 126 | 92.1 | 83.3 | 73.1 |
| PCB 167 | 97.4 | 99.4 | 96.5 |
| PCB 156 | 101.6 | 99.8 | 97.3 |
| PCB 157 | 100.4 | 99.6 | 97.3 |
| PCB 169 | 96.6 | 103.5 | 98.5 |
| PCB 189 | 94.8 | 105.4 | 92.1 |

The range of recovery was from 92.1 % to 102.9 % when DCM/*n*-hexane (2:98, v/v) was used, from 83.3 % to 105.4 % when DCM/*n*-hexane (5:95, v/v) was used and from 73.1 to 105.0 when Ether/*n*-hexane (6:94, v/v) was used.

**Table S4.** Comparison of MDLs obtained by different extraction, cleanup, and detection methods in the previous studies with the ones in this study.

| **Environmental matrix** | **Analytes** | **Extraction** | **Cleanup** | **Analytical instrument**  **(Column)** | **MDL (pg/g)** | **Ref.** |
| --- | --- | --- | --- | --- | --- | --- |
| Soil | 12 dl-PCBs and 17 PCDDs/Fs | Sonication | Multilayer silica gel column  Florisil micro-column | GC-QqQ-MS/MS  DB-5MS (60 m × 0.25 mm × 0.25 μm) | dl-PCBs : 0.16-0.38^a^ PCDDs : 0.25-1.42^a^ PCDFs : 0.21-1.11^a^ | This paper |
| Food and Feed | 12 dl-PCBs and 17 PCDDs/Fs | Accelerated solvent extraction (ASE) | Multilayer silica gel column  Activated carbon column | GC-QqQ-MS/MS DB-5MS (60 m × 0.25 mm × 0.25 μm) | dl-PCBs : 0.13-0.36^b^ PCDDs : 0.018-0.17^b^ PCDFs : 0.025-0.13^b^ | [64] |
| Sediment | 12 dl-PCBs and 17 PCDDs/Fs | Soxhlet  (toluene for 24 hr) | Multi-layer silica gel column Alumina column | HRGC-HRMS Rtx-2330 (60 m × 0.25 mm × 0.1 μm) DB-5MS (60 m × 0.25 mm × 0.25 μm) | dl-PCBs : 0.04-0.29^b^ PCDDs/Fs : 0.004-0.021^b^ | [65] |
| Soil and Egg | 12 dl-PCBs and 17 PCDDs/Fs | Soxhlet  (toluene for 24 hr) | Multilayer silica gel Activated carbon Alumina columns | HRGC-HRMS DB-5MS (60 m × 0.25 mm × 0.25 μm) | dl-PCBs : 0.3 (egg) , 0.1 (soil) PCDDs/Fs : 0.05-0.3 (egg),  0.02-0.1 (soil) | [66] |
| Biological Sample | 17 PCDDs/Fs | QuEChERS | SPE column (6 mm i.d.) | HRGC-HRMS DB-5MS (60 m × 0.25 mm × 0.25 μm) | PCDDs/Fs : 0.25-1.12^b^ | [67] |
| Fly ash | 17 PCDDs/Fs | Soxhlet (24 hr with 250 mL of DCM/n-hexane (3 : 1, v/v)) | Multi-layer silica gel column Alumina column | HRGC-HRMS HP-5MS (60 m × 0.25 mm × 0.25 μm) | PCDDs : 0.2-1.6^a^ PCDFs : 0.1-13.4^a^ | [48] |
| Sediment | 12 dl-PCBs and 17 PCDDs/Fs | Selective pressurized liquid extraction (DCM : hexane =1:1 v/v for dl-PCBs; Toluene for PCDD/Fs) | In-cell clean-up (Silica, alumina, and Florisil were placed in the extraction cell) | GC-ECNI-MS (methane as CI gas)  HRGC-HR/MS  DB-dioxin (60m × 0.25mm × 0.25 μm) | dl-PCBs : 32.2 ± 33.2^a^ PCDDs : 21.9 ± 23.7^a^ PCDFs : 12.5 ± 3.49^a^ | [68] |

^a^ MDL = SD × t (n$-$1, 1$-$α = 0.99), t (6, 0.99) = 3.14 (*n* = 7)

^b^ MDL = SD × 3

**Table S5.** Total WHO-TEQ concentrations of dl-PCBs and PCDDs/Fs for the representative soil sample excavated near industrial complexes.

| **dl-PCBs** | **pg/g** | **pg TEQ/g^a^** | **PCDDs/Fs** | **pg/g** | **pg TEQ/g** |
| --- | --- | --- | --- | --- | --- |
| PCB 81 | 0.357 | 0.000 | 2,3,7,8-TCDF | 0.272 | 0.027 |
| PCB 77 | 0.724 | 0.000 | 2,3,7,8-TCDD | ND (<MDL) | NA |
| PCB 123 | 0.278 | 0.000 | 1,2,3,7,8-PeCDF | 0.437 | 0.013 |
| PCB 118 | 1.593 | 0.000 | 2,3,4,7,8-PeCDF | 2.358 | 0.707 |
| PCB 114 | 0.500 | 0.000 | 1,2,3,7,8-PeCDD | 0.636 | 0.636 |
| PCB 105 | 1.123 | 0.000 | 1,2,3,4,7,8-HxCDF | 3.073 | 0.307 |
| PCB 126 | 0.284 | 0.028 | 1,2,3,6,7,8-HxCDF | 2.096 | 0.210 |
| PCB 167 | 0.286 | 0.000 | 2,3,4,6,7,8-HxCDF | 2.885 | 0.288 |
| PCB 156 | 0.398 | 0.000 | 1,2,3,4,7,8-HxCDD | 0.536 | 0.054 |
| PCB 157 | 0.300 | 0.000 | 1,2,3,6,7,8-HxCDD | ND (<MDL) | NA |
| PCB 169 | 0.496 | 0.015 | 1,2,3,7,8,9-HxCDD | 1.075 | 0.108 |
| PCB 189 | ND (<MDL) | NA^b^ | 1,2,3,7,8,9-HxCDF | 0.646 | 0.065 |
| Σ_12_dl-PCBs (pg/g) | 6.340 |  | 1,2,3,4,6,7,8-HpCDF | 3.330 | 0.033 |
| Σ_12_dl-PCBs (pg TEQ/g) | 0.044 |  | 1,2,3,4,6,7,8-HpCDD | 6.773 | 0.068 |
|  |  |  | 1,2,3,4,7,8,9-HpCDF | ND (<MDL) | NA |
|  |  |  | OCDD | 98.828 | 0.030 |
|  |  |  | OCDF | 3.106 | 0.001 |
|  |  |  | Σ_17_PCDD/Fs (pg/g) | 126.051 |  |
|  |  |  | Σ_17_PCDD/Fs (pg TEQ/g) | 2.547 |  |

^a^ pg TEQ/g : Conc. (pg/g) × WHO_2005_-TEF, ^b^ NA : not applicable

(a) dl-PCBs

(b) PCDDs/Fs


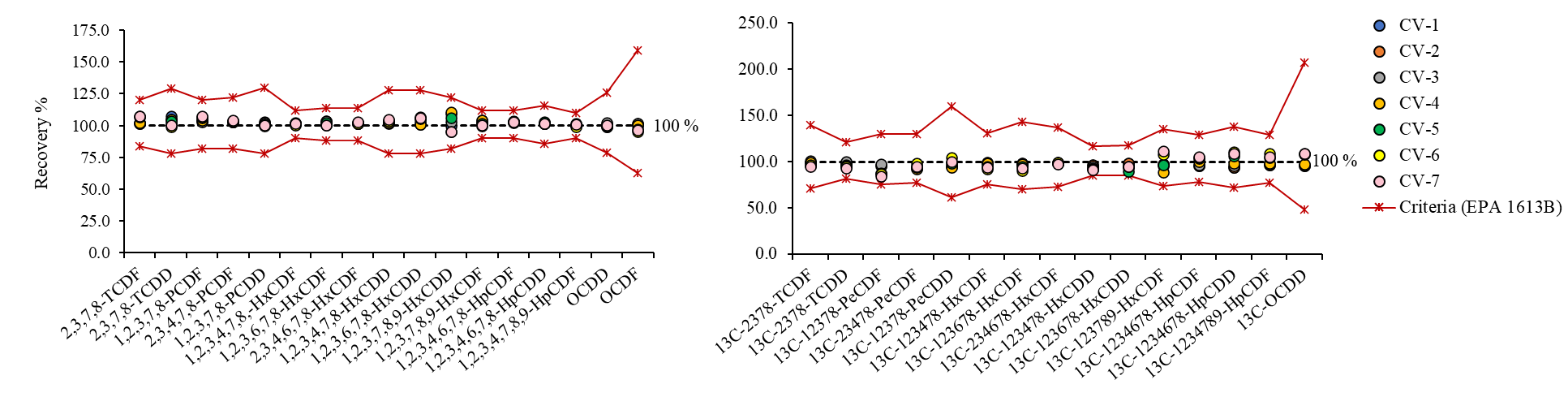


**Figure S1.** Verification charts of (a) dl-PCBs and (b) PCDDs/Fs obtained during sample analysis to monitor the instrumental performance (GC-QqQ-MS/MS).

The WP-CS4 (dl-PCB calibration standard) and CS3 (PCDDs/Fs calibration standard) were used to verify calibration, and the recovery of each congener was calculated. The acceptance criteria followed the requirements of EPA 1668C [69] and EPA 1613B [70] by isotope dilution HRGC/HRMS, and it was found from the results that all congeners satisfied the acceptance criteria.

**Figure S2.** Schematic of the one-step clean-up procedure. .


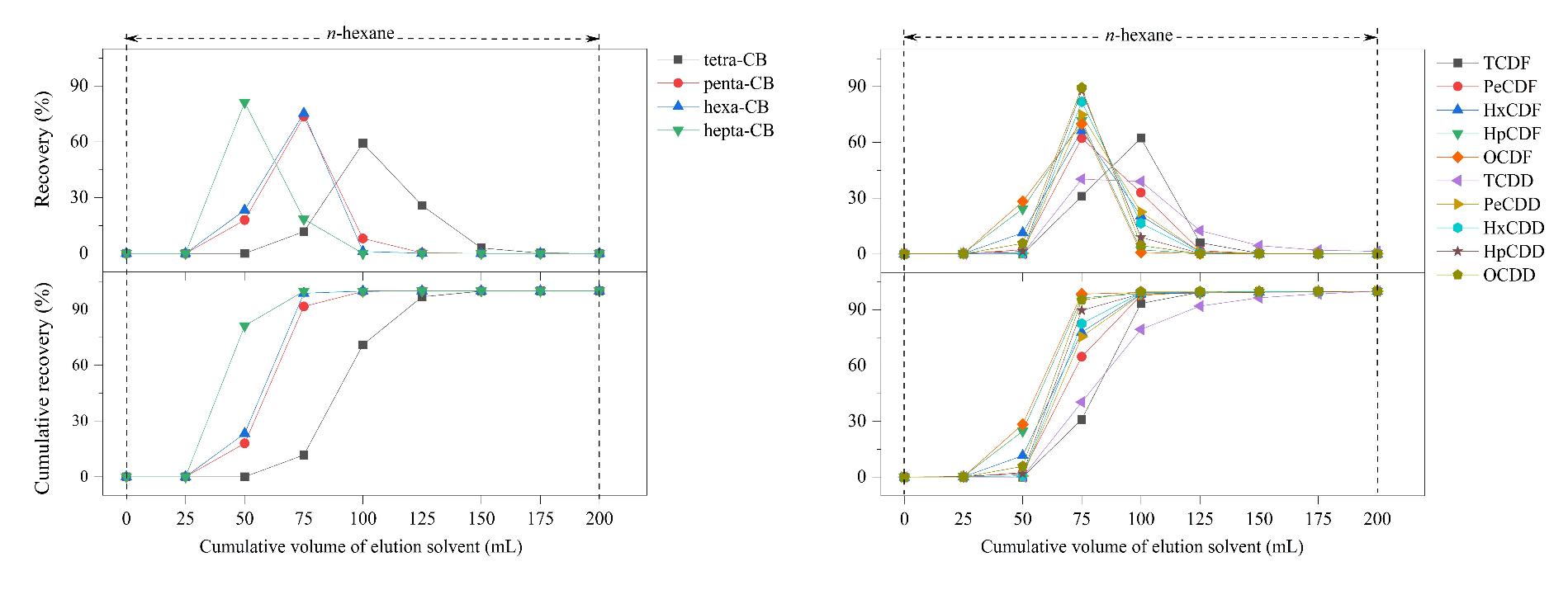


**Figure S3.** Elution profiles of a) dl-PCBs and (b) PCDDs/Fs on the multilayer silica gel column.

DL-PCBs and PCDDs/Fs were eluted with 175 mL of *n*-hexane from the multilayer silica gel column under gravity.

(a)

| a. PCB 81 (Tetra-CB) | b. PCB 77 (Tetra-CB) | c. PCB 123 (Penta-CB) | d. PCB 118 (Penta-CB) | e. PCB 114 (Penta-CB) | f. PCB 105 (Penta-CB) |
| --- | --- | --- | --- | --- | --- |
| g. PCB 126 (Penta-CB) | h. PCB 167 (Hexa-CB) | i. PCB 156 (Hexa-CB) | j. PCB 157 (Hexa-CB) | k. PCB 169 (Hexa-CB) | l. PCB 189 (Hepta-CB) |


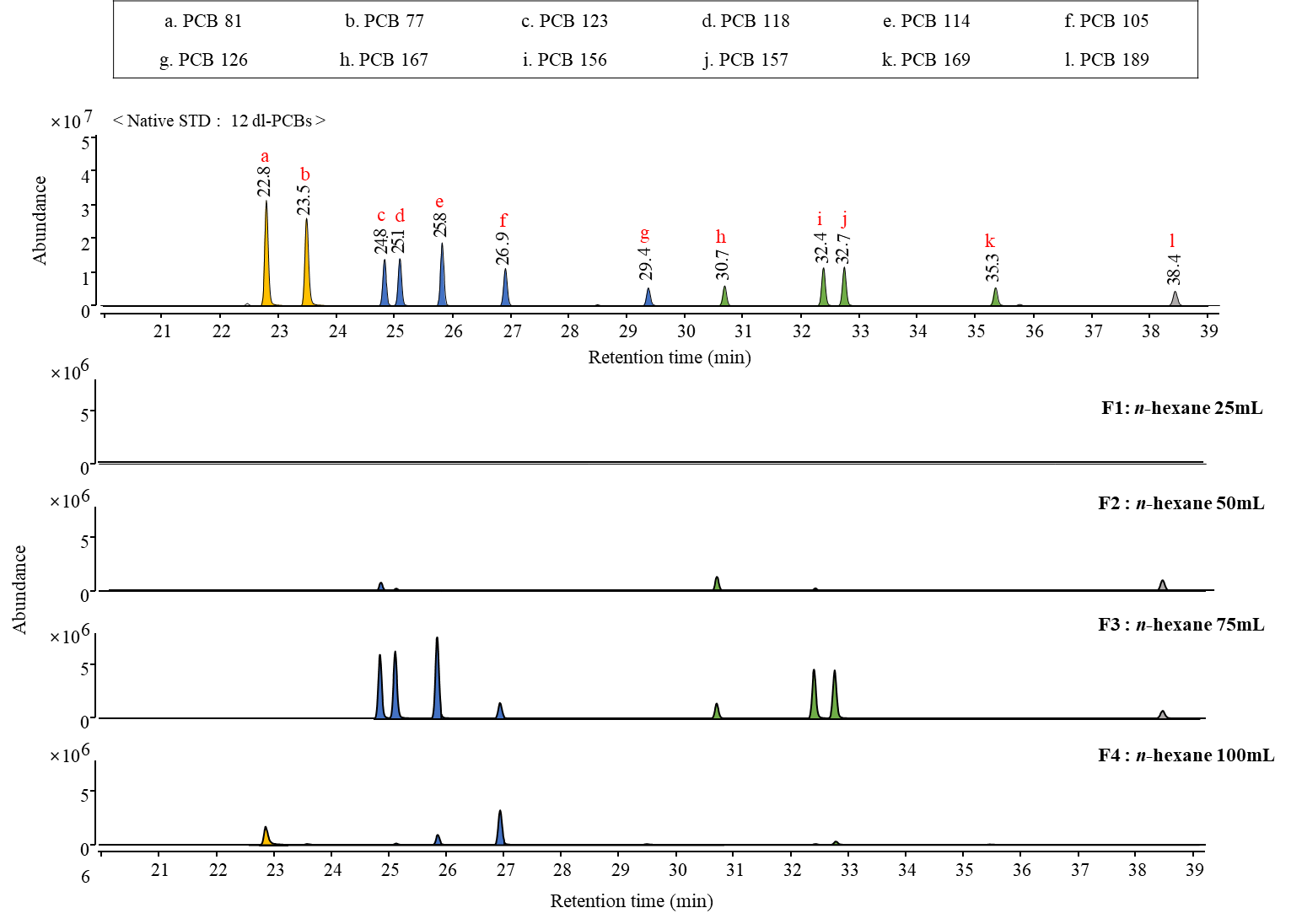


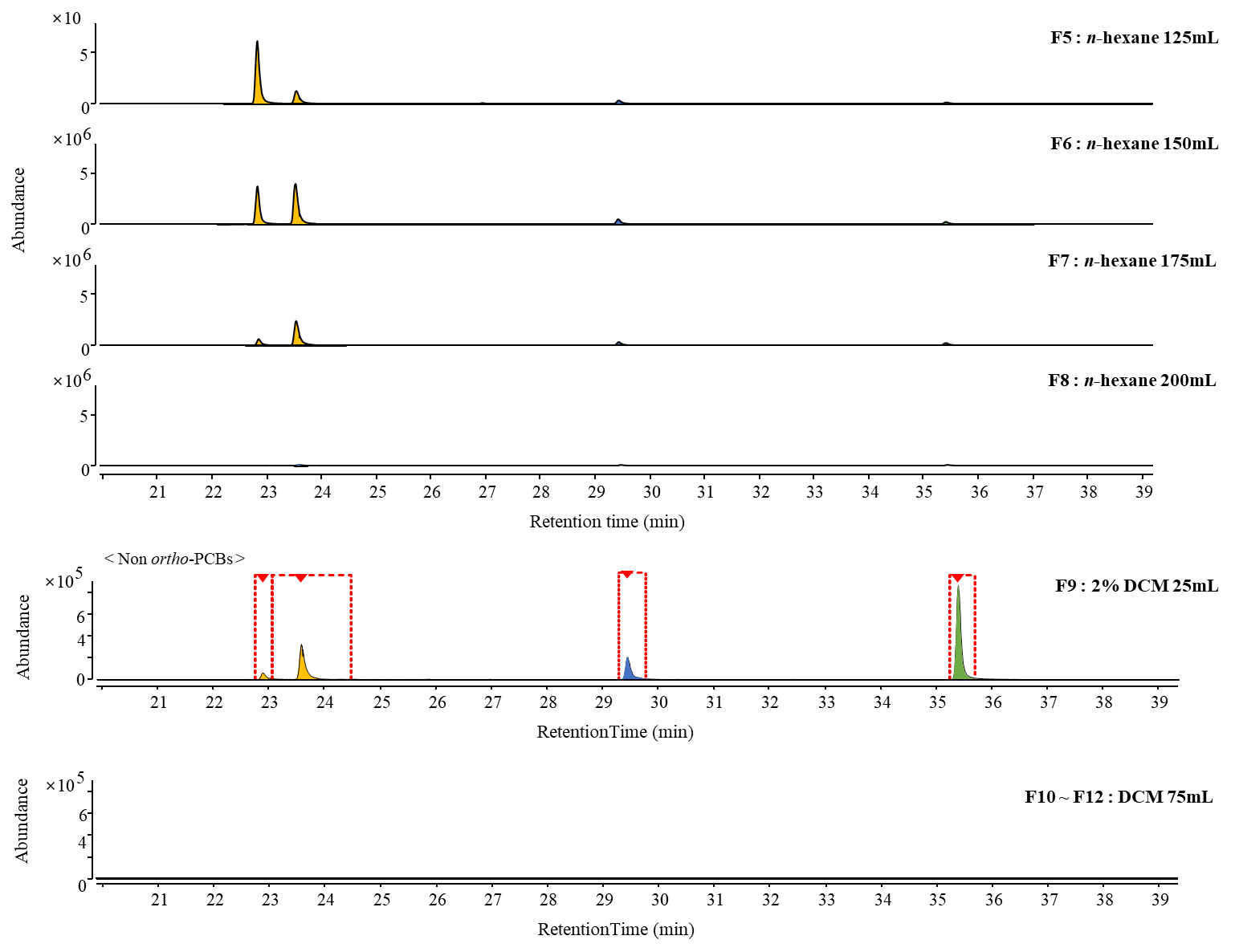


(b)

| a. 2,3,7,8-TCDF | b. 2,3,7,8-TCDD | c. 1,2,3,7,8-PeCDF | d. 2,3,4,7,8-PeCDF | e. 1,2,3,7,8-PeCDD | f. 1,2,3,4,7,8-HxCDF |
| --- | --- | --- | --- | --- | --- |
| g. 1,2,3,6,7,8-HxCDF | h. 2,3,4,6,7,8-HxCDF | i. 1,2,3,4,7,8-HxCDD | j. 1,2,3,6,7,8-HxCDD | k. 1,2,3,7,8,9-HxCDD | l. 1,2,3,7,8,9-HxCDF |
| m. 1,2,3,4,6,7,8-HpCDF | n. 1,2,3,4,6,7,8-HpCDD | o. 1,2,3,4,7,8,9-HpCDF | p. OCDD | q. OCDF |  |


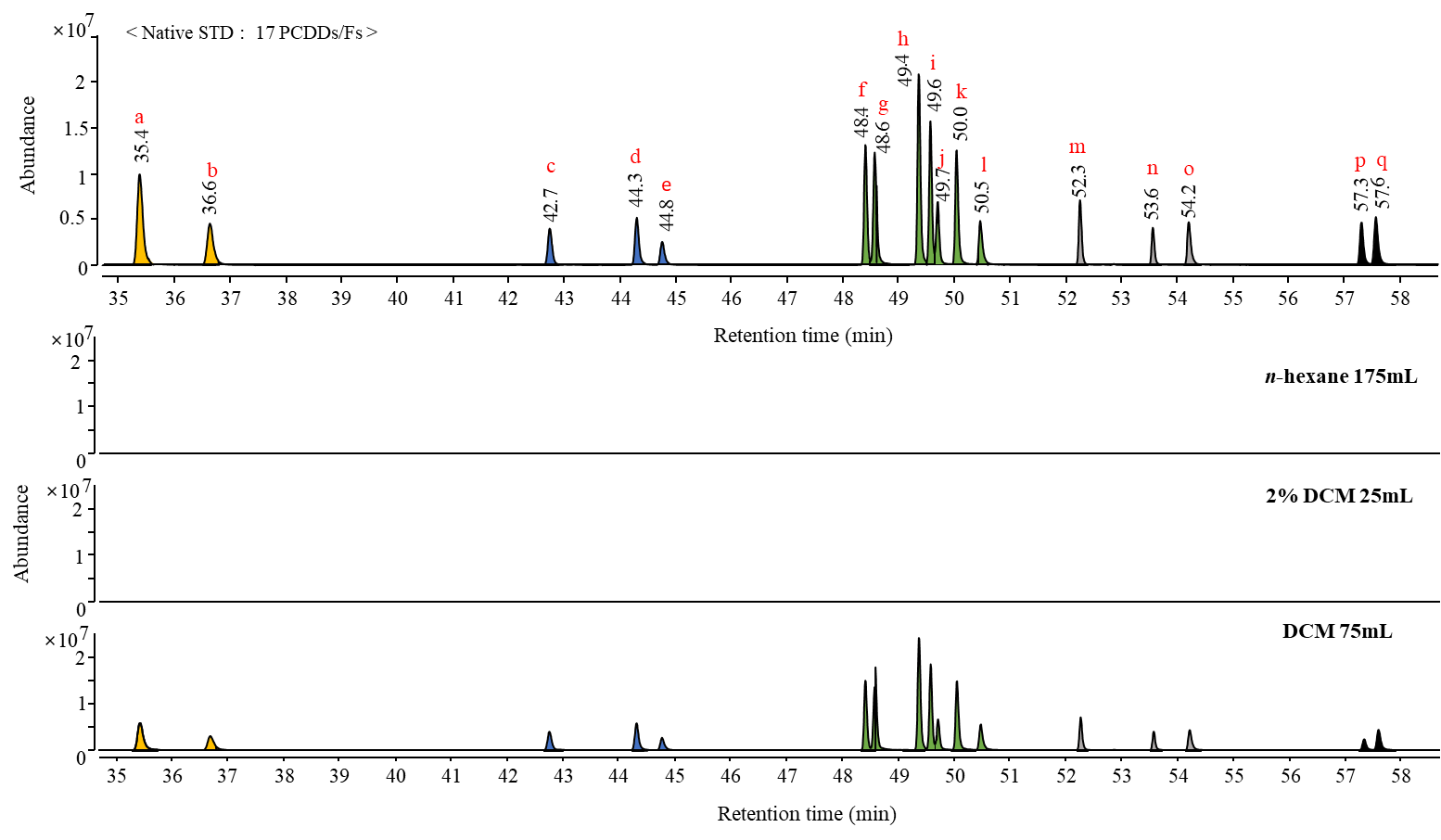


**Figure S4.** Chromatograms of congeners of dl-PCBs and PCDDs/Fs obtained during fractionation.

References

3. Van den Berg, M.; Birnbaum, L.S.; Denison, M.; De Vito, M.; Farland, W.; Feeley, M.; Fiedler, H.; Hakansson, H.; Hanberg, A.; Haws, L. The 2005 World Health Organization reevaluation of human and mammalian toxic equivalency factors for dioxins and dioxin-like compounds. *Toxicol. Sci.* **2006**, *93*, 223-241.

48. Fan, Y.; Zhang, H.; Wang, D.; Ren, M.; Zhang, X.; Wang, L.; Chen, J. Simultaneous determination of chlorinated aromatic hydrocarbons in fly ashes discharged from industrial thermal processes. *Analytical Methods*, **2017**, *9*(35), 5198–5203.

62. Shiu, W.Y.; Doucette, W.; Gobas, F.A.; Andren, A.; Mackay, D. Physical-chemical properties of chlorinated dibenzo-p-dioxins. *Environ. Sci. Technol.* **1988**, *22*, 651-658.

63. Dossier, E. Polychlorinated dibenzo-p-dioxins (PCDDs), polychlorinated dibenzofurans (PCDFs), and dioxin-like polychlorinated biphenyls (dl-PCBs). *Prepared by the sub-group on review of the priority substances list (under Working Group E of the Common Implementation Strategy for the Water Framework Directive, p. 35* **2011**.

64. Sun, H.; Wang, P.; Li, H.; Li, Y.; Zheng, S.; Matsiko, J.; Hao, Y.; Zhang, W.; Wang, D.; Zhang, Q. Determination of PCDD/Fs and dioxin-like PCBs in food and feed using gas chromatography-triple quadrupole mass spectrometry. *Sci. China Chem.* **2017**, *60*, 670–677.

65. Zhang, H.; Zhao, X.; Ni, Y.; Lu, X.; Chen, J.; Su, F.; Zhao, L.; Zhang, N.; Zhang, X. PCDD/Fs and PCBs in sediments of the Liaohe River, China: Levels, distribution, and possible sources. *Chemosphere*, **2010**, *79*(7), 754–762.

66. Nghiem, T. X.; Hoang, A. Q.; Nguyen, T. D.; Nguyen, T. T.; Tran, P. D.; Nguyen, T. T.; Tu, M. B. PCDD/Fs and Dioxin-like PCBs in Chicken Eggs and Soils in Dong Nai Province, Southern Vietnam: Impacts of Raising Methods and Nearby Pollution Sources. *Bulletin of Environmental Contamination and Toxicology*, **2022**, *108*(1), 136–144.

67. Cloutier, P.-L.; Fortin, F.; Groleau, P. E.; Brousseau, P.; Fournier, M.; Desrosiers, M. QuEChERS extraction for multi-residue analysis of PCBs, PAHs, PBDEs and PCDD/Fs in biological samples. *Talanta*, **2017**, *165*, 332–338.

68. Aguilar, L.; Williams, E. S.; Brooks, B. W.; Usenko, S. Development and application of a novel method for high-throughput determination of PCDD/Fs and PCBs in sediments. *Environmental Toxicology and Chemistry*, **2014**, *33*(7), 1529–1536.

69. Method 1668C. Chlorinated Biphenyl Congeners in Water, Soil, Sediment, Biosolids, and Tissue by HRGC, *USEPA* **2010**.

70. Method 1613, Revision B: Tetra‐through octa‐chlorinated dioxins and furans by isotope dilution HRGC/HRMS. *UESPA* **1994**.
